# Pure White Cell Aplasia Associated With Long-Term Unprotected Exposure to High Concentrations of Benzalkonium Chloride and 2-Phenoxyethanol

**DOI:** 10.7759/cureus.49473

**Published:** 2023-11-27

**Authors:** Mathias Lutz, Daniel T Neumann, Francisco Farfán López, Tim Pfeiffer, Klaus Hirschbühl

**Affiliations:** 1 Hematology and Oncology, University of Augsburg, Augsburg, DEU; 2 Pathology, University of Augsburg, Augsburg, DEU

**Keywords:** pure white cell aplasia, agranulocytosis, neutropenia, benzalkonium chloride, 2-phenoxyethanol, upper respiratory tract infection

## Abstract

Pure white cell aplasia (PWCA) is a very rare hematological disorder with a nearly total absence of granulocytes and their precursor cells. While the disease is rarely diagnosed incidentally in otherwise asymptomatic individuals, most patients suffer from sometimes life-threatening infections. Due to its very low incidence, the precise pathomechanism of PWCA still needs to be elucidated. While most cases reported in the literature have been associated with an underlying thymic or autoimmune disease, some other factors including the intake of certain drugs such as antimicrobial agents or immune checkpoint inhibitors have been identified as potential triggers. Since PWCA is commonly refractory to treatment with granulocyte colony-stimulating factors (G-CSF), the main focus lies in identifying and eliminating the underlying trigger.

Here, we report a unique case where the development of PWCA in a 56-year-old man with an upper respiratory tract infection has to be attributed to the long-term unprotected exposure to an industrial detergent containing high concentrations of the preservatives benzalkonium chloride (BAC) and 2-phenoxyethanol (2-PE). As a matter of fact, certain hematotoxic potential has been described in the literature for both BAC and 2-PE.

## Introduction

Pure white cell aplasia (PWCA) is a very rare hematological condition characterized by agranulocytosis with a total absence of myeloid precursor cells in bone marrow whereas erythropoiesis and megakaryopoiesis usually remain preserved [1,2]. While the disease is rarely diagnosed as an incidental finding in otherwise asymptomatic individuals, most patients suffer from recurrent infections. Due to the very low incidence of PWCA, its precise pathomechanism still needs to be elucidated [3]. However, in most of the cases described in the literature, an association with thymoma or other thymic disorders, with autoimmune diseases or with the intake of certain drugs, has been identified [1,2,4-8]. Up to now, no standard therapy regimen for PWCA has been established, and it is, furthermore, commonly refractory to treatment with granulocyte colony-stimulating factor (G-CSF) [2]. Therefore, the main focus lies on eliminating the underlying trigger.

Benzalkonium chloride (BAC) has a wide spectrum of antimicrobial activity against gram-positive and gram-negative bacteria, fungi, protozoa, and even some enveloped viruses [9]. Due to its additional ability to facilitate transcorneal drug delivery, it is one of the most used preservatives in ophthalmic solutions. Furthermore, it is found in various other liquid medications such as nasal sprays or bronchial inhalers [10]. Existing evidence favors the view that BAC is generally tolerated well at the low doses encountered in the pharmaceutical preparations mentioned [11].

Likewise, 2-phenoxyethanol (2-PE) provides a broad spectrum of antimicrobial activity against gram-positive and gram-negative bacteria and yeasts [12]. On the other hand, it exerts only weak inhibitory effects on the physiological local skin flora [13]. Hence, in low concentrations, it is considered safe for people of all ages including children and accordingly has been widely used as a preservative, especially in cosmetics and fragrances, for decades [12,13].

However, toxic systemic effects have been described at higher levels of exposure to both substances mentioned mainly in in vitro studies [10,14]. Handling of BAC and 2-PE in higher concentrations is, therefore, recommended only with the use of protective measures [15].

To the best of our knowledge, we here report the first case of PWCA development following long-term unprotected exposure to an industrial detergent containing high concentrations of both BAC and 2-PE.

## Case presentation

A 56-year-old male patient with fever, signs of an upper respiratory tract infection, and severe agranulocytosis was admitted to our hospital after initial treatment at a peripheral facility.

At the presentation at our hospital, he reported fatigue and a cough that had first appeared one week earlier. Due to the additional development of fever, he presented to his local hospital five days ago. There, laboratory tests revealed significant leukopenia and elevation of C-reactive protein (CRP), and the patient was admitted for intravenous antibiotic therapy with piperacillin/tazobactam. Further laboratory work-up yielded isolated agranulocytosis leading to referral to our hospital as mentioned.

The patient denied dyspnea, chest pain, night sweats, weight loss, and changes in urination or defecation. He occasionally drank alcohol and was a long-time smoker with a consumption of about 10 cigarettes per day over a period of about 40 years. Except for an oral vitamin D supplementation, he explicitly denied consumption of any other medication, herbal supplements or illicit or illegal drugs. The patient was intellectually disabled since his birth but had no other preexisting conditions, especially no known cardiac diseases. Furthermore, he never had a documented episode of leukopenia or neutropenia before. He always lived in Germany and never undertook longer journeys to foreign countries. He denied unprotected sexual intercourse and any unusual contact with animals or lentic water. The patient worked in the scullery of a local restaurant for decades and lived in an assisted living facility due to an intellectual disability.

Initial physical examination revealed a significantly damaged skin of both hands from the wrist to all fingertips. In these areas, the skin was erythematous and scaly with partially open, partially bloody crusted lesions. Apart from this, there were no anomalies. In particular, no lymphadenopathy or splenomegaly was palpable. Laboratory tests on admission at our hospital showed moderate CRP elevation and leukopenia with nearly absolute loss of neutrophils (absolute neutrophil count of 0.04×10^3^/μL). Hemoglobin was only mildly reduced and platelets were slightly above the upper reference range. All other laboratory tests including electrolytes, renal, hepatic, thyroid, and coagulation parameters were normal (Table 1).

**Table 1:** Results of routine laboratory tests acquired from the patient at admission and discharge Reference ranges mentioned in the table are used at the University Hospital of Augsburg, Germany, and apply to male adults without medical conditions affecting the results. CKD-EPI, chronic kidney disease epidemiology collaboration

Laboratory parameter	Reference range	Admission	Discharge
Complete blood count			
Leukocytes (×10^3^/μL)	3.0-10.0	1.76	4.09
Erythrocytes (×10^6^/μL)	4.5-6.1	3.93	4.29
Hemoglobin (g/dL)	14.0-18.0	11.9	12.6
Hematocrit (%)	42.0-52.0	36.1	38.4
Mean corpuscular volume (fL)	82.0-101.0	91.9	89.5
Mean corpuscular hemoglobin (pg)	27.0-34.0	30.3	29.4
Mean corpuscular hemoglobin concentration (g/dL)	31.5-36.0	33.0	32.8
Platelets (×10^3^/μL)	140-440	464	434
Manual white blood cell differential count			
Band neutrophils (%)	0-5	0	0
Segmented neutrophils (%)	50-70	2	1
Lymphocytes (%)	20-50	78	75
Monocytes (%)	0-15	13	16
Eosinophils (%)	0-10	2	0
Basophils (%)	0-3	5	1
Smudge cells (%)	n/a	0	7
Metabolic parameters			
Sodium (mmol/L)	136-145	138	138
Potassium (mmol/L)	3.5-5.1	3.68	4.09
Creatinine (mg/dL)	0.7-1.2	0.79	0.86
Estimated glomerular filtration rate (CKD-EPI formula; mL/min/1.73m²)	>90	>90	>90
Lactate dehydrogenase (U/L)	0-250	150	163
C-reactive protein (mg/dL)	0-0.5	2.70	0.53
Liver function parameters			
Total bilirubin (mg/dL)	0-1.2	0.27	n/a
Alanine aminotransferase (U/L)	10-50	36	n/a
γ-glutamyltransferase (U/L)	0-60	36	n/a
Alkaline phosphatase (U/L)	40-130	71	n/a
Butyrylcholinesterase (U/L)	5320-12920	7552	n/a
Blood coagulation parameters			
Prothrombin time (%)	82-125	97	n/a
International normalized ratio	0.9-1.15	1.01	n/a
Partial thromboplastin time (s)	26-36	28	n/a

In view of the cough and fever, a chest X-ray was performed but showed no abnormalities. Extensive microbiological examination of several blood cultures as well as sputum and urine revealed no pathogens. The above-mentioned intravenous broad-spectrum antibiotics were continued and an oral antiviral and antifungal prophylaxis with aciclovir and fluconazole was added. Furthermore, stimulation with G-CSF (over a total duration of 13 days) was initiated. Over the next few days, this treatment resulted in an appropriate decrease in clinical and laboratory infection signs. However, no effect on the leukopenia could be noted.

**Figure 1:**
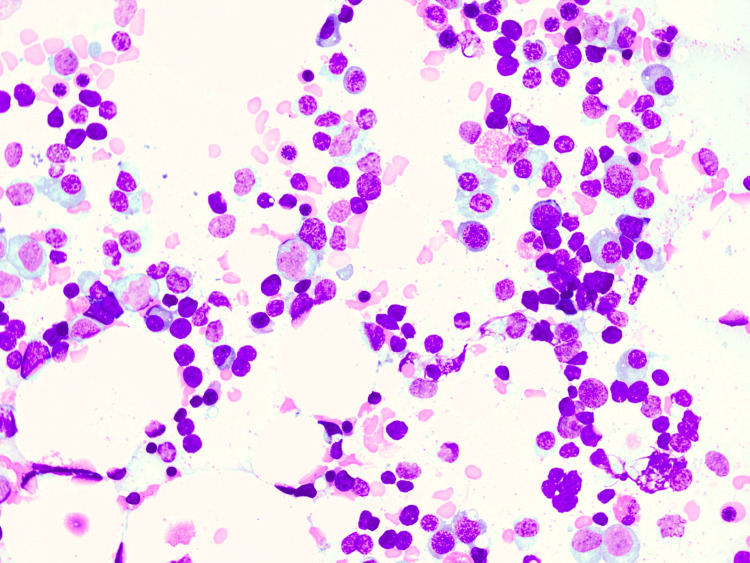
Bone marrow aspirate Bone marrow aspirate (Pappenheim stain, original magnification ×40) of the patient demonstrated a markedly hypocellular marrow with a nearly total absence of granulocytes whereas erythropoiesis and megakaryopoiesis are largely unimpaired.

**Figure 2:**
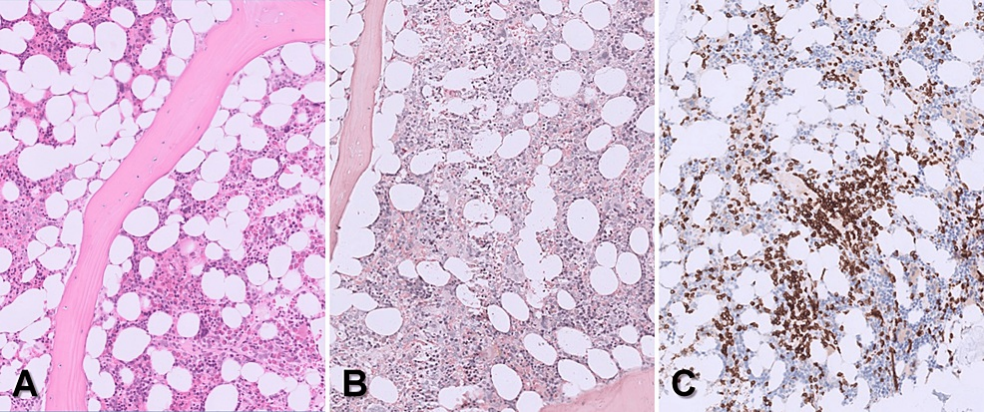
Bone marrow biopsy Bone marrow biopsy of the patient shows normal spongiosa and age-appropriate reduced fat content (A: Hematoxylin and eosin stain, original magnification ×5), while the marrow’s microarchitecture is nearly completely missing (B: Chloroacetate esterase staining, original magnification ×5) and T cells are diffusely increased (C: CD3 staining, original magnification ×5).

Subsequently, a bone marrow biopsy was performed. Cytological and histological analysis revealed the characteristic picture of PWCA with a total absence of myeloid cells (neither mature cells nor precursors) whereas erythropoiesis and megakaryopoiesis were unremarkable (Figure 1 and Figure 2). Flow cytometry identified a small T cell population with an aberrant immunophenotype (CD3+, CD2-, CD5+, CD7+, CD4+, CD8-, CD16+, CD52-, CD30-). Hence, a clonality analysis of the T cell receptor beta and gamma loci was performed but remained negative. Further bone marrow diagnostics were unremarkable. In total, a toxic or immunological cause of bone marrow damage had to be assumed.

At this point, we questioned the patient and his legal guardian again to identify a potential causative agent of PWCA. However, no new aspects arose from this conversation. Taking into account the finding of PWCA and recommendations from the available literature, the patient thereupon underwent extensive immunological, metabolic, virological, and microbiological testing. Serum immunofixation revealed an unspecific immunoglobulin G lambda band pair against the polyclonal background. Further immunological analyses showed borderline positivity for the anti-nuclear antibody (ANA) but otherwise were negative. All other laboratory tests mentioned above were unremarkable. These results are summarized in Table 2.

**Table 2:** Comprehensive immunological, metabolic, microbiological, and virological evaluation performed to identify the underlying cause of PWCA during the patient’s hospitalization Reference ranges mentioned in the table are used at the University Hospital of Augsburg, Germany, and apply to male adults without medical conditions affecting the results. DNA, deoxyribonucleic acid; EBNA, Epstein-Barr virus nuclear antigen; EIA, enzyme immune assay; ELISA, enzyme-linked immunosorbent assay; IIF, indirect immunofluorescence; IgG, immunoglobulin G; IgM, immunoglobulin M; IgA, immunoglobulin A; PCR, polymerase chain reaction

Laboratory parameter	Reference range	Result
Immunoglobulins		
IgA (mg/dL)	70-400	349
IgG (mg/dL)	700-1600	1800
IgM (mg/dL)	40-230	160
Autoimmune/inflammatory disorders		
Rheumatoid factor (IU/mL)	0-14	<10
Anti-neutrophil cytoplasmic antibody (IIF)	Negative	Negative
Anti-nuclear antibody (IIF)	<1:100	1:200
Anti-double stranded DNA antibody (ELISA; IU/mL)	0-100	<10
Complement C3 (mg/dL)	90-180	148
Complement C4 (mg/dL)	10-40	38.8
Anti-thyroglobulin antibody (IU/mL)	<72	16
Anti-thyroid peroxidase antibody (IU/mL)	<34	13
Anti-thyrotropin receptor antibody (IU/L)	<1.75	1.2
Vitamins and trace elements		
Transferrin (mg/dL)	200-360	203
Transferrin saturation (%)	16-45	13.6
Soluble transferrin receptor (mg/L)	1.71-4.13	2.7
Ferritin (ng/mL)	30-400	594
Folate (ng/ml)	3.9-26.8	9.6
Vitamin B12 (pg/mL)	191-663	670
25-OH vitamin D (ng/mL)	20-70	31
Reticulocytes		
Reticulocytes (absolute; pL)	n/a	0.056
Reticulocytes (relative; %)	0.5-2.0	1.43
Reticulocyte hemoglobin equivalent (pg)	n/a	31.7
Virological parameters		
Hepatitis A virus (IgM)	Negative	Negative
Hepatitis B virus (surface antigen)	Negative	Negative
Hepatitis B virus (core antibody)	Negative	Negative
Hepatitis C virus (EIA)	Negative	Negative
Hepatitis E virus (antibody)	Negative	Negative
Human immunodeficiency virus 1/2 (antibody)	Negative	Negative
Human immunodeficiency virus (p24 antigen)	Negative	Negative
Cytomegalovirus (IgG; IU/mL)	<0.5	0.4
Cytomegalovirus (IgM)	Negative	Negative
Cytomegalovirus DNA, quantitative (IU/mL)	Negative	Negative
Epstein-Barr virus (EBNA IgG; U/mL)	<22	136
Epstein-Barr virus DNA, quantitative (IU/mL)	<180	Negative
Herpes simplex virus (IgG; relative unit/mL)	<16	195.4
Herpes simplex virus (IgM)	Negative	Negative
Herpes simplex virus 1 DNA (copies/mL)	<33	Negative
Herpes simplex virus 2 DNA (copies/mL)	<120	Negative
Human herpesvirus 6A PCR (IU/mL)	<425	Negative
Human herpesvirus 6B PCR (IU/mL)	<140	Negative
Parvovirus B19 (IgG; ratio)	<1.1	<0.1
Parvovirus B19 (IgM; ratio)	<1.1	<0.1
Parvovirus B19 PCR (IU/mL)	<41	Negative
Varicella zoster virus (IgG; IU/L)	<80	433
Varicella zoster virus (IgM)	Negative	Negative
Varicella zoster PCR (copies/mL)	<10	Negative
Microbiological parameters		
Chlamydia pneumoniae (IgG)	Negative	Negative
Chlamydia pneumoniae (IgA)	Negative	Negative
Mycoplasma (IgG; U/mL)	<20	11.3
Mycoplasma (IgM; ratio)	Negative	Negative
Mycoplasma (IgA; ratio)	Negative	Negative

Furthermore, a contrast-enhanced computed tomography (CT) scan of the neck, chest, abdomen, and pelvis was performed and showed no abnormalities. In particular, there was no evidence of thymoma, splenomegaly, or lymphadenopathy. The cardiac evaluation also was unremarkable.

Finally, we discussed all these findings again with the patient and his legal guardian. During this conversation, it actually turned out that the patient had been working with a potentially toxic industrial detergent in the above-mentioned scullery for many years without wearing protective gloves (which were actually recommended). On reviewing the ingredients contained therein, it became apparent that these included BAC and 2-PE in high concentrations. After studying the literature, we were able to elicit the possible hematotoxic potential of these two substances.

In the synopsis of the clinical, laboratory, pathological, and imaging results, an association between PWCA and long-term unprotected exposure to the industrial detergent seemed at least very likely. Therefore, we discussed in detail with the patient and his legal guardian our recommendation to refrain from using the aforementioned agent in the future or at least to consistently wear protective gloves. After a total hospitalization of about three weeks, the patient could be discharged home. Since he was still neutropenic at discharge, antimicrobial prophylaxis was continued.

Follow-up over the following weeks and months showed the patient in good condition. He credibly denied any new exposure to the industrial detergent and the former skin damage of both hands was completely resolved. At the last presentation ten months after the above-mentioned inpatient treatment, neutrophils were completely normalized.

## Discussion

PWCA is a very rare condition that has been described in the literature mainly anecdotally. Accordingly, as of now, no specific incidence can be defined and the pathomechanism of the disease has hardly been elucidated [1,3]. However, in review of the existing literature, there is widespread consensus that an immunological process compromising the granulopoiesis is likely to underlie the disease. This assumption is supported by two findings: on the one hand, in vitro studies could show the inhibition of colony-forming units of granulocytes and macrophages by serum obtained from PWCA patients suggesting the existence of immunological mechanisms against myeloid precursor cells [1,16]. On the other hand, most cases of PWCA are associated with either a disorder of the thymus, and thus the hotbed of the immune system, or with several autoimmune diseases [1,2,4-6]. However, in other cases, there was no such predisposition, instead viral infections or the consumption of various drugs could be identified as the triggering cause [17]. Among the latter, most cases have been described after the intake of antimicrobial or anti-inflammatory agents [7,8]. Table 3 gives an overview of potential causes of PWCA as described in the literature.

**Table 3:** Overview of potential causes of PWCA as described in the literature PWCA, pure white cell aplasia

Category	Examples described in the literature	References
Thymic disorders	Thymoma	[1,2]
	Good syndrome	[18]
	Thymic carcinoma	[4]
Autoimmune diseases	Autoimmune hepatitis	[5]
	Primary biliary cirrhosis	[6]
	Systemic sclerosis	[3]
	Autoimmune thyroiditis	[2]
Drugs	Anti-infective agents (e.g., imipenem, amodiaquine)	[7,8]
	Anti-inflammatory agents (e.g., ibuprofen, mesalazine)	[8]
	Immune checkpoint inhibitors (e.g., durvalumab)	[19]

However, no such underlying conditions could be identified in the case reported here. Other relevant potential causes of neutropenia could be ruled out by reviewing the patient's medical history and all examination findings. For example, Barth syndrome could be excluded when taking into account the patient's unremarkable cardiac status. Immune neutropenia, on the other hand, was unlikely considering the long-term follow-up without further administration of G-CSF [20]. In the end, we were able to identify the patient's long-term exposure to an industrial detergent as the only reasonable PWCA trigger. In addition to some ingredients for which no such potential is described, the detergent contained BAC and 2-PE.

Both of these substances exhibit broad antimicrobial activity against various bacteria and fungi, and to some extent also against protozoa and enveloped viruses. Since both BAC and 2-PE have shown to be well tolerated at low doses, they are widely used as preservatives in commercially available products such as cosmetics and fragrances but also in pharmaceuticals including eye drops, nasal sprays, or bronchial inhalers [10-13].

However, studies have shown a certain toxic potential of BAC and 2-PE at higher concentrations. For example, Boston and colleagues performed a trial investigating the in vitro and in vivo effects of nasal saline spray containing BAC on human neutrophils [10]. For this purpose, they incubated neutrophil granulocytes in vitro with either different concentrations of the nasal saline spray or with phosphate-buffered saline and then compared cell viability, morphological changes, and activity of lactate dehydrogenase (LDH) as a marker for cell lysis between the two groups. Furthermore, healthy volunteers were exposed to the BAC-containing nasal saline spray or the buffered saline without preservatives in vivo and their neutrophil granulocytes were analyzed afterward. In both experiments, no effects were seen after the use of buffered saline. However, the authors could demonstrate a decrease in cell viability, an alteration of morphological structure, and an increase in LDH activity of neutrophil granulocytes after exposure to the nasal saline spray containing BAC in a time- and concentration-dependent manner. Accordingly, they concluded that ready-to-use nasal saline spray preparations exhibit toxic effects on human neutrophils and that particularly BAC is responsible for this toxicity [10].

On the other hand, Starek et al. could show the hemolytic potential of 2-PE by treating male Wistar rats with different doses of 2-PE subcutaneously for four weeks [14]. While a time- and dose-dependent decrease of erythrocytes and hemoglobin could been shown, leukocyte counts were significantly reduced only at the highest dose of 2-PE. The results of this study support the aforementioned fact that 2-PE can be considered safe at lower doses but has a remarkable toxic potential at higher concentrations [14,15].

Taken together, and in the absence of other explanations, our findings suggest that in the case described here, PWCA was caused by long-term unprotected exposure to the industrial detergent containing BAC and 2-PE, among others. Therefore, this unique case report is intended to draw attention to the hematotoxic potential of both these substances especially when they are used in higher concentrations without protective measures.

As PWCA is rarely diagnosed incidentally in otherwise asymptomatic patients but usually becomes apparent by an infectious disease, anti-infective treatment has to be given the first priority. Taking into account the significant neutropenia, antibiotic treatment is usually supplemented by antifungal and antiviral prophylaxis in these cases. Subsequently, the main focus lies on eliminating the underlying causes, especially since PWCA characteristically shows no response to G-CSF [2,5]. Treatment is difficult in patients with autoimmune diseases since an immunosuppressive regimen has to be initiated despite the already existing immunodeficiency [3,5,18]. If a thymoma can be identified as a PWCA trigger, it should be resected if possible [1,2]. Finally, if a causative agent such as a drug could be identified, it should be omitted immediately. If the initial infection can be treated successfully and the underlying cause can be eliminated, PWCA usually recovers completely.

## Conclusions

PWCA is a very rare condition characterized by the almost complete absence of neutrophils and, therefore, often associated with infectious complications. While existing data suggest that the disease is associated primarily with thymic or autoimmune disorders, other underlying causes occasionally need to be considered.

In the case reported here, the chemical substances BAC and 2-PE were identified as the most likely cause of PWCA. Due to their good tolerability in low concentrations, these two substances are widely used as preservatives in liquid medications, cosmetics, and fragrances, among others. In higher concentrations, however, BAC and 2-PE obviously show a remarkable toxic potential, so they, like in this case, should only be used with appropriate protective measures.
